# Comparison between a wireless dry electrode EEG system with a conventional wired wet electrode EEG system for clinical applications

**DOI:** 10.1038/s41598-020-62154-0

**Published:** 2020-03-23

**Authors:** Hermann Hinrichs, Michael Scholz, Anne Katrin Baum, Julia W. Y. Kam, Robert T. Knight, Hans-Jochen Heinze

**Affiliations:** 1 Department of Neurology, Otto-von-Guericke University, Leipziger Str. 44, 39120 Magdeburg, Germany; 2 Department of Behavioural Neurology, Leibniz Institute of Neurobiology, Brenneckestr. 6, 39120 Magdeburg, Germany; 3 Center for Behavioural Brain Sciences, Otto-von-Guericke University, Universitätsplatz 2, 39106 Magdeburg, Germany; 4 German Centre for Neurodegenerative Diseases, Otto-von-Guericke University, Leipziger Str. 44, 39120 Magdeburg, Germany; 5 Helen Wills Neuroscience Institute, University of California – Berkeley, 132 Barker Hall, Berkeley, CA 94720 USA; 6 Department of Psychology, University of California – Berkeley, 130 Barker Hall, Berkeley, CA 94720 USA; 7 Forschungscampus STIMULATE, Magdeburg, Germany

**Keywords:** Cognitive ageing, Stroke, Epilepsy

## Abstract

Dry electrode electroencephalogram (EEG) recording combined with wireless data transmission offers an alternative tool to conventional wet electrode EEG systems. However, the question remains whether the signal quality of dry electrode recordings is comparable to wet electrode recordings in the clinical context. We recorded the resting state EEG (rsEEG), the visual evoked potentials (VEP) and the visual P300 (P3) from 16 healthy subjects (age range: 26–79 years) and 16 neurological patients who reported subjective memory impairment (age range: 50–83 years). Each subject took part in two recordings on different days, one with 19 dry electrodes and another with 19 wet electrodes. They reported their preferred EEG system. Comparisons of the rsEEG recordings were conducted qualitatively by independent visual evaluation by two neurologists blinded to the EEG system used and quantitatively by spectral analysis of the rsEEG. The P100 visual evoked potential (VEP) and P3 event-related potential (ERP) were compared in terms of latency, amplitude and pre-stimulus noise. The majority of subjects preferred the dry electrode headset. Both neurologists reported that all rsEEG traces were comparable between the wet and dry electrode headsets. Absolute Alpha and Beta power during rest did not statistically differ between the two EEG systems (p > 0.05 in all cases). However, Theta and Delta power was slightly higher with the dry electrodes (p = 0.0004 for Theta and p < 0.0001 for Delta). For ERPs, the mean latencies and amplitudes of the P100 VEP and P3 ERP showed comparable values (p > 0.10 in all cases) with a similar spatial distribution for both wet and dry electrode systems. These results suggest that the signal quality, ease of set-up and portability of the dry electrode EEG headset used in our study comply with the needs of clinical applications.

## Introduction

The quality of scalp electroencephalogram (EEG) recordings critically depends on the connection between the amplifier input and the skin surface. Wet electrodes that rely on conductive gel to guarantee low impedance levels (<10 KOhm) remain the gold standard for clinical recordings. However, EEG recording with wet electrodes requires skin abrasion, gel application, impedance optimization and cleaning after recording, all of which are time consuming. Trained EEG technicians are therefore recommended for wet electrode EEG set-up and acquisition (for instance, see^1–3^). However, this presents a barrier to realising diagnostic strategies that propose replacing EEG recordings in the clinic or the doctor’s office with EEG recordings in the patient’s home, which saves both time and costs as well as improving the patient’s health care and comfort^4,5^.

To achieve reliable home-based EEG recording, electrodes must be easy to apply and provide stable data quality over long recording sessions. The same standards hold for other applications, including repeated EEG recordings in the context of neurorehabilitation. Regarding the first key issue, dry electrodes do not require skin abrasion, gel application, or a trained technician; therefore, they might be useful for EEG recording in the home setting. In contrast, wet electrodes may take longer to apply, and their set ups are not well tolerated by patients over longer recording periods in part because of the tension and discomfort exerted by the elastic chin straps that are tightly connected to hold the EEG cap in place. Regarding the second issue, wet electrodes tend to have reduced signal quality after several hours when the conductive gel dries out, failing to meet the stability of data quality requirement^1,2,6,7^. For longer recording periods, a modified version of wet electrodes is usually applied, in which the electrodes are glued on the skin using collodion. This technique maintains the signal quality over long periods of time, provided that electrode conductant is periodically refilled^8^. However, for practical concerns, this procedure is even less suitable for home recordings compared to conventional wet electrodes. In contrast, given that dry electrodes do not require gel application, their signal quality is maintained over longer periods of time.

Dry electrode EEG systems have recently been proposed (see^2^ for a review and^9–11^ for a comparison of various devices with respect to research applications) as an alternative both for clinical and home monitoring usage, as well as applications in real life situations such as during sports^3,12,13^. Most of these developments apply a bundle of one and up to more than 20 conductive pins per electrode where the pins are coated by either silver, gold or nickel^1,3,6,14–22^. Most developments were motivated by brain computer interface (BCI) concepts rather than by clinical needs. The majority of the cited devices - except for the systems presented in^1,6,22^ - did not include all 21 electrodes of the 10–20 system, which is required for clinical applications. The International Federation of Clinical Neurophysiology^23,24^, defined the anatomical locations of these 21 electrodes uniformly covering the human scalp, and recommended this set up for clinical EEG recordings. Another practical consideration of dry electrode utilization is its certification by the *Communauté Européenne (CE)*, an organization that certifies a medical device if and only if it complies with the regulations of the European Union. This certification is mandatory for medical application but is not mentioned in the aforementioned publications.

Given that dry electrodes are placed on the skin without any gel application, the dry EEG system typically results in larger impedances compared to wet EEG systems with electrodes that require conductive gel^25,26^. To date, few studies have directly compared the data quality between these two systems^6,11,18,21,27^. The question remains whether the signal quality of dry electrodes used in our study can match the quality of wet electrodes in the context of clinical applications, i.e. involving neurological patients. If so, this would support clinical, in office and home monitoring applications of the multi-channel dry EEG system used in our study.

Here, we compare a newly designed CE certified dry electrode EEG head set with integrated wireless data transmission with a traditional wet and wired electrode EEG recording system used in routine clinical conditions. Resting state EEG (rsEEG) as well as two types of event-related potentials (ERPs) were recorded from a group of healthy volunteers as well as patients who reported subjective memory impairment (SMI) and were referred from a dementia clinic to the Magdeburg Neurological Clinic. The comparison of the wet and dry EEG systems included subjects’ report of their comfort level with the headsets, percentage of artifactual segments excluded from analyses, a visual evaluation of the rsEEG by experienced neurologists blinded to the EEG systems used, quantitative spectral power measures of the rsEEG, as well as amplitude and latency measures of the ERPs.

In order to ensure that the dry EEG electrode system measures what it was designed to measure, namely EEG, we tested the validity of this system by comparing its signal quality to the state-of-the-art wet EEG system. Comparable signal quality between the two systems provides evidence for construct validity of the dry EEG system. This is an important prerequisite for establishing support for future clinical, office, and home monitoring usage. We hypothesized that (i) the dry electrode EEG system’s signal quality was comparable to the conventional clinical EEG recording system with wet electrodes both in healthy volunteers and in neurological patients in all the recorded measures, including eyes closed rsEEG activity and tasks generating ERPs, and (ii) healthy volunteers and neurological patients accepted the dry EEG system at least as much as the conventional wet EEG system.

## Methods

### Subjects

Sixteen subjects who reported being healthy at the time of recording (age = mean: 42.3 years, range: 26–79 years) and 16 patients (age = mean: 71.0 years, range: 50–83 years) were included in the study. The patients reported subjective memory impairment (SMI) but - except two - did not meet the Mini Mental State Examination (MMSE) score for Mild cognitive Impairment. Comorbidities were not reported. Healthy subjects were recruited from the students and staff of Magdeburg University and from relatives of the SMI patients. SMI-patients were referred from a Dementia Clinic to the Neurological Department. Subjects were only included if they were able to understand the consent process. No further exclusion criteria were applied. The current experiment was conducted as part of a clinical study and was approved by the local ethics committee of Otto-von-Guericke University. All subjects provided informed consent. Details in visual and auditory acuity were not available but all participants were able to read the information sheet and understand verbal instructions.

### Experimental procedures

All recordings were conducted in the same room at the Neurological department of the Magdeburg University at approximately the same time of the day (right before or after noon). Each recording session included a sequence of four components, with subjects sitting in an upright position: resting state EEG (rsEEG) with eyes open (2 min, to get the subjects familiarized with the recording situation), rsEEG with eyes closed (5 min), visual attention task that elicited the P100 visual evoked potential (VEP) and visual target detection task that elicited the P3 ERP component. Each subject participated in two recording sessions, one using the conventional wet and wired electrode headset and a second using the dry and wireless electrode headset. The sequence of the two sessions was randomized and counterbalanced across subjects, with a maximum of one week between recording sessions.

All recordings were done by the chief medical technical assistant of the neurological university clinic with decades of clinical EEG experience (in recording both rsEEG and evoked potentials). Subsequently, all EEG were visually checked and evaluated by EEG neurologists with extensive clinical EEG-experience. The signal quality was further checked by an automated artifact detection procedure as described below.

Barry *et al*.^28^ and Staba^29^ showed that eyes open rsEEG primarily reflects cortical processing of visual input. These processes may vary between the two recording sessions and thus lead to variations not attributable to the type of EEG system. Therefore, we only present results from the rsEEG recorded during the eyes closed condition.

In addition, we also report the time it took to mount the two types of headsets including electrode placement.

#### EEG recording using wet electrodes

An Inomed PL231 clinical EEG recorder (Inomed Medizintechnik GmbH; Emmendingen, Germany) was used for a referential EEG recording from all 19 Ag/AgCl passive electrodes based on the international 10–20 system (FP1, FP2, F7, F3, Fz, F4, F8, T3, C3, Cz, C4, T4, T5, P3, Pz, P4,T6, O1 and O2 according to Jasper and colleagues (1958)^24^ plus bilateral mastoids that where placed on the left and right earlobe. The reference/ground electrode was placed close to Cz/Fpz. Electrode impedances were kept below 5 KOhm in all recordings and electrode sites. The input impedance of the EEG amplifier was> 100 MOhm. All signals were low pass filtered with 90 Hz cut off (−3dB) frequency and digitized with a sampling rate of 256 Hz (16 bit resolution, least significant bit (LSB) 0.5 µV, noise with shortened inputs <2.5 uV peak-to-peak).

**Figure 1:**
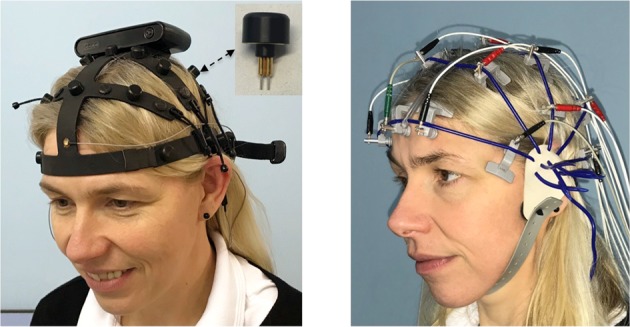
EEG headsets. Left: Dry and wireless EEG system: F1 headset with Ag electrodes. The module on top of the headset contains all units to process, store and transmit the EEG signals. The insert shows the dry electrode as mounted at each of the 19 10–20 electrode sites. Right: Wet and wired EEG system: Inomed recording net with Ag/AgCl electrodes.

For electrode placement and EEG recording, the subjects were seated in a comfortable chair. A commercial EEG cap (rubber net, also known as Schröter cap, see Fig. 1) was used to manually place each of the wet electrodes according to the anatomical locations according to the 10–20-electrode system (Ref. ^24^ see^30^ for further details). The EEG technician selected the best cap fitting the individual subject’s head from three possible sizes. Following recommendations^31^, an average precision of about 4 mm can be expected with this procedure compared to coordinates determined by a laser optic procedure in that study.

#### EEG recording using dry electrodes

The newly developed CE certified dry EEG headset F1 (Nielsen TeleMedical, Magdeburg/Germany) consists of 19 dry electrodes magnetically attached to a headset that is connected to a module, as shown in Fig. 1. Informed consent was obtained from the subject shown in this figure for publication with identifying information or images in an online open-access publication. This module includes a board with amplifier and digitization electronics, which allows for wireless signal transmission to a base station; however it can alternatively store up to 24 hours of EEG data on an embedded flash memory chip, making full mobility possible in the home recording environment.

The recording system consists of dry electrodes with two spring-loaded silver pins per electrode (see also^19,22,32^ for similar solutions), The pins are available in two different lengths (12 and 15 mm) to accommodate different head shapes and hair volume, thereby avoiding the need of a chin strap. The two-pins per electrode set up is in line with findings of a previous study^32^ that systematically evaluated various dry electrode designs differing in the number of pins (referred to as ‘fingers’ in their paper) per electrode. These authors concluded “that sparser arrangements of fingers are more robust to varying use cases and are more effective at penetrating through hair on the scalp”.

EEG was recorded from the 19 above mentioned conventional 10–20 dry electrodes positions, plus additional Ag electrodes on the left and right mastoids that were placed on the scalp (i.e. behind but not on the earlobes) using a disposable sticker. The ground and reference electrodes were located near Fpz. At each electrode site (except for mastoids), a double spring-loaded silver pin recorded the EEG signals. The amplifier’s input DC impedance is 500 MOhm, consistent with the high electrode impedances expected of dry electrodes, and with the average impedance of approximately 500 KOhm recorded in this study (see results). In order to minimize environmental noise disturbing data acquisition (for instance, nearby moving objects), the headset is completely passively shielded. In addition, the system is equipped with an active feedback loop via the ground electrode. After analog low pass filtering (95 KHz cut off frequency) and oversampling with 1 MHz/channel the signals were digitally low pass filtered with 130 Hz cut off (−3dB) frequency and finally down sampled to 500 Hz/channel (digital resolution 24 bit, LSB 0.04 µV, noise with shortened inputs <2.0 µV peak-to-peak).

Dry electrode placement and recording was performed using the same chair as previously mentioned. The F1 dry electrode headset was mounted on the subject’s head by the same EEG technician that performed the wet EEG recordings. The F1 headset is available in three different sizes to accommodate various head sizes. Once it is applied, the array of relative electrode positions is predefined by the corresponding frame holding the electrodes, thereby avoiding misplacement of individual electrodes. However, a systematic error of a few mm affecting all electrodes can happen, which may also occur with wet electrode caps with fixed electrode locations.

#### Questionnaire to assess subjects’ acceptance of the EEG headsets

All subjects were asked to assess the comfort level and usability of the wet and dry EEG systems. For that purpose they filled out a written questionnaire after the recording. As documented in Table 1, the majority of subjects were in favour of the F1 dry EEG headset. This holds for the approximate 20 min recording per patient and headset (including breaks between the four components). However, given that a few subjects expressed discomfort about the pointiness of the pins in the dry electrodes at the end of the recording, the question arose whether in the case of longer recording periods (as is expected in home recordings) the wet electrode headset would be advantageous. To address this, we recruited an additional 22 patients and 20 healthy volunteers (overall mean age 46.7 years, 25 females) and asked them to wear both the dry and wet electrodes headset/cap with the electrodes for one hour (as applied by the same experienced technician in all cases). After 20, 30 and 60 minute intervals, subjects rated the comfort level on a Likert scale ranging from 1 (unbearable) to 7 (did not notice it). The level of comfort regarding the application of the two headsets was also documented along the same scale. The two headsets were applied on separate days within a week at about the same time of day. The sequence was randomized and balanced across the two groups of subjects. On this occasion, we measured the time to apply the headsets including the electrodes preparation.

**Table 1:** Subjects’ responses to the questionnaire.

Questions	Response
Have you undergone any EEG examination in the past?	23 yes
	6 no
	3 did not provide an answer
Which type of electrodes would you prefer?	23 dry electrodes.
	4 both equal
	3 wet electrodes
	2 did not provide an answer
How comfortable is the F1 dry EEG headset?	26 Headset fits well
	4 Too much pressure
	2 Afraid they would lose electrodes in case of head movements
Would you be able to apply the headset by yourself?	22 yes
	8 yes, with another person’s support
	1 no
	1 did not provide an answer

The possible answers provided by the questionnaire corresponded to the answers listed in the right column.

#### Visual target detection task (P3 ERP)

**Figure 2:**
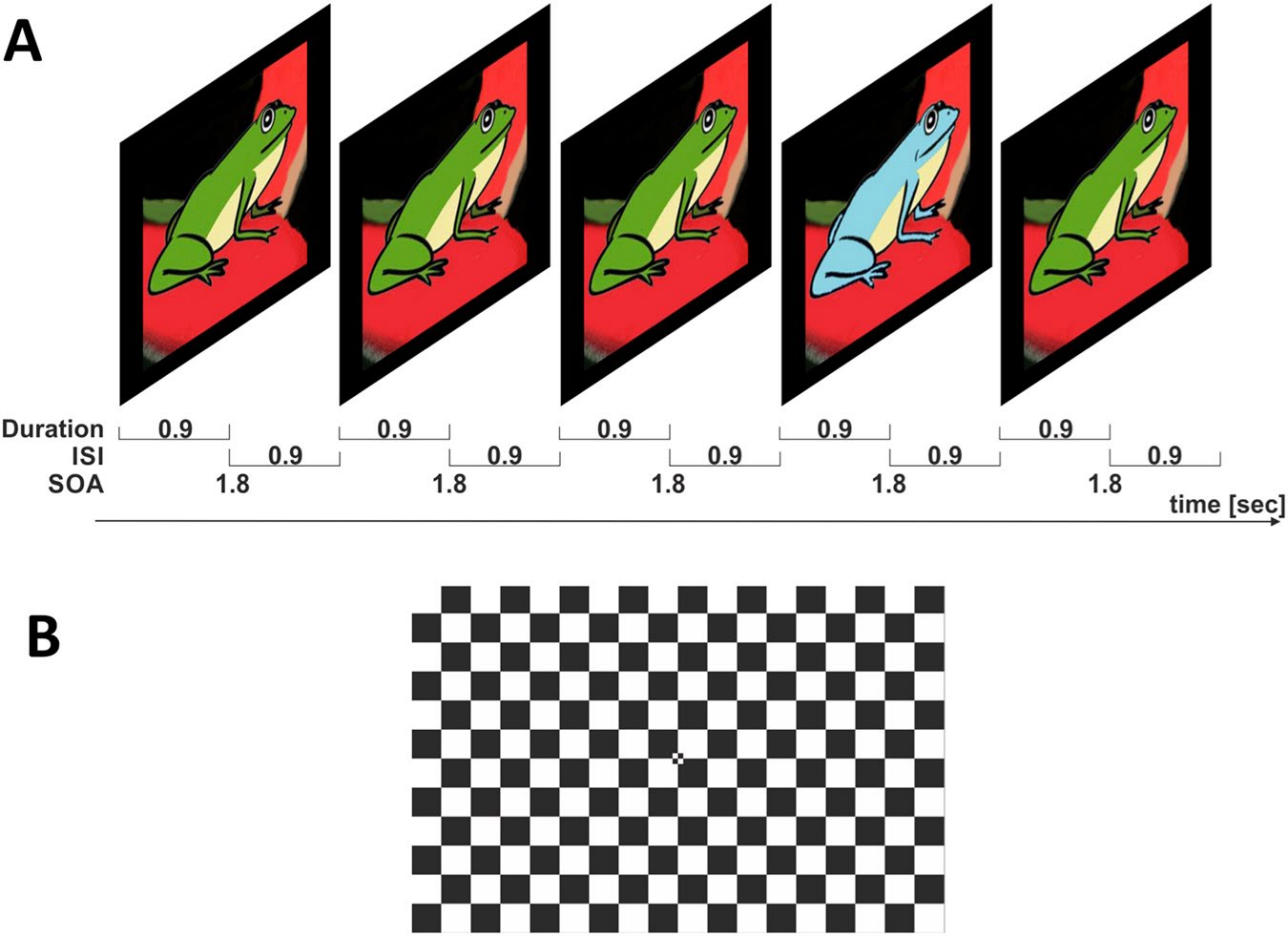
(**A**) Target detection task (P3) paradigm: Green/blue frog represents the standard/target stimulus with 80/20% frequency of occurrence. Frog images by courtesy of Nielsen Consumer Neuroscience. ISI = inter stimulus interval; SOA = stimulus onset asynchrony. (**B**) Visual attention task: Checker board stimulus to elicit a P100 VEP.

Subjects performed a visual target detection experiment. In the target detection paradigm, a random sequence of 60 blue or green frogs (horizontal viewing angle 7.5 degrees) was presented to the subjects with frequencies of 20% (blue/target stimulus) and 80% (green/standard stimulus). See Fig. 2A for an illustration of the experimental paradigm. The blue and green frog stimuli used were compliments of Nielsen Consumer Neuroscience^33^.

Subjects were instructed to press a computer mouse button when they saw the target: a blue frog. The button press must occur within 100–800 ms after stimulus onset in order to be included in subsequent analyses. Stimulus duration was 0.9 sec with 1.8 sec stimulus onset asynchrony (SOA). The task lasted 108 seconds. As patients were generally not able to endure longer experimental tasks, we opted for this short version of a target detection task. For comparability, we implemented the same duration for both patients and controls.

#### Visual attention task (P1 VEP)

A 18 × 12 rectangle grid of alternating black and white squares in checker board pattern (horizontal angle of the entire screen 26.5 degrees, see Fig. 2B) with the black and white colour inverting every 0.6 sec (i.e. SOA = 0.6 sec) was presented on a computer screen to elicit the P1 VEP. This is the most commonly used task in clinical settings to elicit a VEP. Subjects were instructed to keep their eyes on a fixation cross, which was located in the centre of the screen. The task comprised of 200 pattern reversals and lasted 120 seconds.

### Data processing

All numerical processing was done using Matlab version R2015b (*The Mathworks*).

All methods were performed in accordance with the relevant guidelines and regulations.

#### Re-referencing

Resting state EEG (rsEEG) data was re-referenced to a common average reference based on all 19 electrodes in the 10–20 position. For both visual tasks, EEG data was re-referenced to the average of the T3 and T4 electrodes. P3 ERPs are usually referenced to the mastoid electrodes^34^. However, given that in our experiment, the mastoid electrode locations slightly differed between the two recording systems (as described above), systematic differences could occur between the two systems if we had used the mastoid electrodes as reference. Therefore, we changed this standard reference to the average of the T3/T4 electrodes, which resulted in a slight change in the P3 topography. Importantly, this referencing scheme was implemented in both systems, rendering the results comparable.

#### Artifact detection and spectral analysis

Before running any artifact detection or removal procedure, each EEG trace was submitted to a high pass filter at 1 Hz and a notch filter at 50 Hz and 100 Hz to remove line noise. Next, artifacts were identified by a threshold criterion applied to the difference signal (i.e. sum of absolute differences (SAD)) computed over a temporal window of 0.5 sec and a threshold 8 mV/sec. These artifacts could disturb the EOG removal procedure (see below) and were therefore replaced by zeros for this procedure only, after which values of the original signal were restored.

Eye blinks were identified by similarity in shape and topography with a predefined fixed template. No such artifacts occurred in the rsEEG recorded during eyes closed. An epoch of 1500 ms was centered around each EOG event increasing the dominance of the EOG compared to the underlying EEG. A minimum noise fraction (MNF) transform^35,36^ was applied to all EEG channels of that epoch. The MNF transform outputs a set of components that vary in their signal-to-noise ratio, where noise in this case reflects EEG. After removing the component with the largest signal-to-noise ratio, the inverse MNF leads to the original signal with the EOG-artifact largely removed.

Next, spectral measures were applied to identify artifacts not captured by the preceding methods. For that purpose a spectral analysis was conducted according to the Welch^37^ procedure. Data were segmented into two-second epochs with 50% overlap. Each segment was windowed with a Bartlett (=triangle) function. Spectral decomposition was carried out by means of the Fast Fourier Transform (FFT) algorithm. Absolute spectral band power values were computed for the following frequency bands: Delta1 (1–1.5 Hz), Delta2 (1.5–4 Hz), Theta (4–8 Hz), Alpha (8–13 Hz), Beta (13–30 Hz), Gamma1 (30–47 Hz), Gamma2 (53–95 Hz). The frequency bands Delta1 was included to capture slow fluctuations and the two Gamma bands were included to capture high frequency noise. These frequency bands were included only for artifact detection purposes and were not relevant for clinical applications; therefore, we focus our subsequent spectral analyses on Delta2, Theta, Alpha and Beta. Next, for each frequency band, the median of these power values was determined over all segments and all channels. An epoch of a channel was labeled as artifactual and excluded from subsequent analyses if its band power fell below 0.1*median or exceeded the value of 20*median of the respective frequency band.

Finally, segments (rs EEG) or epochs (EP) were rejected as artifacts if the absolute amplitudes exceeded a threshold of 5.5 times the standard deviation computed across the entire recording in each channel of the respective task.

#### Susceptibility to 50 Hz line noise

To compare the susceptibility of the two recording systems to line noise, we averaged the power spectrum of the rsEEG over the frequency range of 49–51 Hz.

#### Calculation of absolute spectral band power values of rsEEG

In a second step, spectral analysis of the rsEEG was repeated, applying the same procedure as described above, however removing all segments identified as artifacts by the aforementioned criteria and omitting the high pass and notch filtering mentioned above. Absolute spectral band power values were computed for the following frequency bands, which are typically reported in clinical EEG settings: Delta (1.5–4 Hz), Theta (4–8 Hz), Alpha (8–13 Hz), Beta (13–30 Hz).

#### Analysis of visual target detection (P3) and attention (VEP) task

The P3 ERP component was extracted from all artifact-free epochs with correct trials only, taking the difference between the averaged response to target and standard stimuli. Epoch length was 1200 ms including a 500 ms pre stimulus interval. As a result of using the T3/T4-reference (as opposed to the standard mastoid reference), the largest P3 amplitude was observed at O1 and O2 rather than in central midline sites. The P3 peak latency was derived from the waveform observed by averaging across all subjects, over the O1 and O2 electrode locations and over both recording systems (dry/wet electrodes). P3 amplitudes at O1 and O2 for each recording system were determined by taking the average of the P3 waveform amplitude over the latency range from 350 to 440 ms, representing an interval symmetrically centered around the peak latency of 395 ms.

The P100 VEP was extracted by averaging all artifact-free epochs and subtracting the pre-stimulus baseline amplitude. Epoch length was 700 ms including a 200 ms pre stimulus interval. The latency of the P100 VEP was derived from the waveform observed by averaging over all subjects, the O1 and O2 electrode locations and both recording sessions (with dry and wet electrodes). P100 amplitudes at O1 and O2 for each recording were determined by taking the average of the P100 waveform amplitude over the latency range from 100 to 130 ms, representing an interval symmetrically centred around the peak latency of 115 ms.

#### Visual evaluation by clinical neurologists

All rsEEG recordings (Inomed and F1) were visually evaluated by two clinical neurologists with long standing EEG experience who were blinded to the EEG system employed. They were asked to report both the type of spontaneous or background EEG activity as well as potential pathological EEG signs, guided by the recommendation of the German Society for Clinical Neurophysiology (DGKN)^38^.

#### Statistical analyses

We conducted statistical comparisons of the aforementioned outcome measures between the wet and dry EEG systems by means of Wilcoxon’s sign rank test, as this non-parametric test does not assume normal distributions. In particular, spectral band power values do not meet this assumption^39^. Accordingly, in the results section we report the z-value of the approximating normal distribution associated with the sign rank test and its corresponding p-value. Multiple comparisons were corrected for by the false discovery rate (FDR) procedure as proposed by^40^.

## Results

Here we present rsEEG results from all 32 participants and ERP-results from only 31 subjects. One subject had to be excluded from the ERP analyses as she did not complete the full set of tasks in the second recording session.

### Time to apply the headsets

The time needed to mount the headset on the subjects’ head for the dry EEG headset (mean/median/standard deviation (SD)) = 4.02/4.00/0.7 min) was significantly shorter than the time needed for the wet EEG headset (mean/median/SD = 6.36/6.35/1.18 min; Z = −5.51, p < 0.00001).

#### Example recording

**Figure 3:**
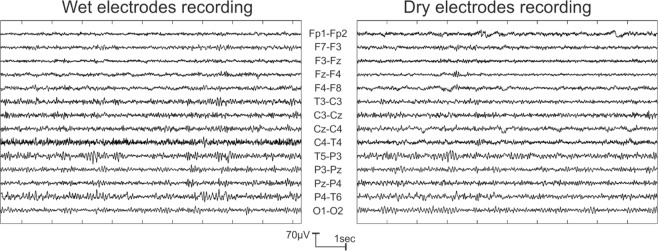
Representative EEG traces (bipolar montage) of the two systems recorded from the same subject during resting state with eyes closed.

A representative example of a 10 second period from the same subject recorded by the wet and dry EEG system is shown in Fig. 3.

#### Questionnaire on comfort and usability

The comfort and usability of the two systems obtained after each recording session are reported in Table 1.

The comfort level during electrode preparation and while wearing the two headsets as reported by a separate group of 42 subjects is shown in Table 2. The difference in comfort level after wearing the headsets for 30 min and 60 min was statistically significant, with subjects favouring the dry system.

**Table 2:** Subjects’ Likert-scale rating of subjective comfort during electrode preparation and while wearing the headsets. SD = standard deviation.

Comfort Level Rating	Dry electrodesn (mean/median/SD)	Wet electrodes (mean/median/SD)	Statistical comparison
During electrode preparation	5.83/6/1.34	5.64/6/1.37	*Z* = 0.87, *p* = 0.390
After wearing the headsets for 20 min	5.75/6/1.08	5.42/6/1.13	*Z* = 1.82, *p* = 0.068
After wearing the headsets for 30 min	5.69/6/1.02	4.54/6/0.73	*Z* = 4.14, *p* = <0.001
After wearing the headsets for 60 min	5.07/5/0.63	3.24/3/0.35	*Z* = 4.94, *p* < 0.001

#### Impedances

**Figure 4:**
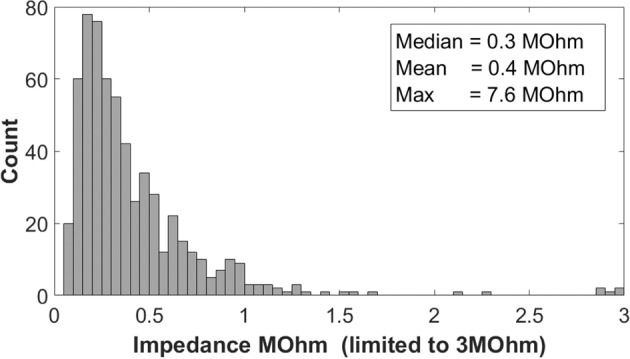
Histogram of dry electrode impedance values observed at all electrodes and all subjects.

A histogram of impedance values across all electrodes and subjects for the dry electrodes recorded during rsEEG is shown in Fig. 4.

The mean/median/maximum impedance values of the F1 headset across all 19 electrodes and all subjects were 0.4/0.31/7.6 MOhm. Impedance values were below 0.5 MOhm in 74% of cases. The impedances of the wet EEG recordings were not documented in the EEG data files and therefore they are not reported here. Nevertheless, the technician ensured the impedance level of all electrodes were under 5 KOhm, which is well below the EEG amplifier’s input impedances of 100 MOhm so that the signal quality was not compromised by the electrode impedances.

#### Artifacts

##### rsEEG

**Figure 5:**
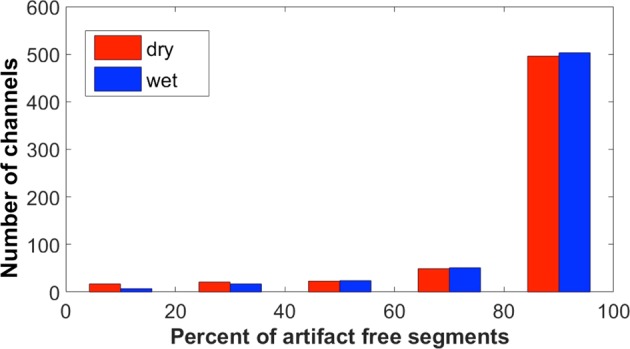
Number of rsEEG-channels (as observed over all 608 channels across 32 participants) with different percentages of artifact-free 2 sec epochs according to the automatic procedure. Separate bars represent the following ranges 0–20%, 20–40%, 40–60%, 60–80%, 80–100%.

The mean/median/SD percentage of artifact-free segments as determined by the automatic artifact detection algorithm per channel and recording session was statistically larger for the wet (88.1/89.9/8.7%) relative to the dry (85.6/88.3/11.8%) EEG systems (*Z* = −2.3, *p* = 0.023), as shown in Fig. 5.

#### P1 VEP and P3 ERP

The mean/median/SD percentage of artifact-free epochs was lower for the dry EEG system for both the P100 VEP and P3 ERP (P100: wet 95.5/97.3/4.3% vs. dry 88.5/92.8/14.4%, *Z* = −3.60, *p* < 0.001,; P3: wet 92.2/93.5/5.9% vs. dry 83.3/89.4/17.3%, Z = −2.90, p = 0.004).

#### rsEEG: Visual evaluation by experienced clinical neurologists

All complete EEG recordings in both wet and dry EEG sessions were rated as ‘interpretable’ by both neurologists; in other words, the data did not contain excessive artifacts or other disturbances that might mask the key physiological EEG features. Out of the 31 subjects, 23 were categorized as Alpha-type, six as partial Beta (i.e. Alpha that was occasionally interrupted or superimposed by Beta activity), one as Beta-type and one as irregular-type of EEG. This classification of each subject’s data was identical for the wet and dry EEG systems, thus resulting in maximal inter rater reliability as reflected by a Cohens Kappa value of 1.0. Importantly, both experienced neurologists agreed in all cases regarding this classification of the spontaneous background activity. No pathological EEG activity was reported in either wet or dry recording sessions.

#### rsEEG: Spectral analysis and band power values

**Figure 6:**
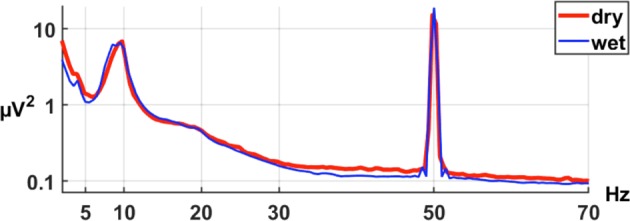
Power spectrum averaged across all electrode sites and subjects for the dry (red line) and wet (blue line) electrode system.

The two recordings were conducted within one week under the same conditions to reduce intra-individual variability^41–43^. As shown in Fig. 6, the power spectrum averaged across all electrode sites and all subjects was comparable between the systems. For each frequency band (Delta, Theta, Alpha and Beta), the absolute power values for the wet and dry EEG systems correlated (Spearman’s rank correlation) positively with each other (p < 0.0001 in all cases).

The pairwise statistical comparison of all these band power values are shown in Table 3. There are no significant differences between EEG systems, except in the Theta-band (only in medium and high impedances ranges) and the Delta band (in all impedance ranges) where greater power was observed with the dry electrodes.

**Table 3:** Statistical evaluation of differences in band power values between dry and wet electrodes at different dry electrode impedance ranges.

Frequency band	Statistical comparison (Z/p)		
	Impedance < 0.31 MOhm	Impedance 0.31–1.0 MOhm	Impedance 1.0–8 MOhm
Delta, 1.5–4.0 Hz	*Z* = −7.80, *p* < 0.0001	−11.50/<0.0001	−4.90/<0.0001
Theta, 4.0–8.0 Hz	−1.65/0.097	− 3.56/0.0004	−3.36/0.0008
Alpha, 8.0–13.0 Hz	1.40/0.14	1.95/0.0503	−0.03/0.97
Beta, 13.0–30.0 Hz	0.50/0.62	0.06/0.95	1.48/0.14

The median value of all dry electrode impedances was 0.31 MOhm.

**Figure 7:**
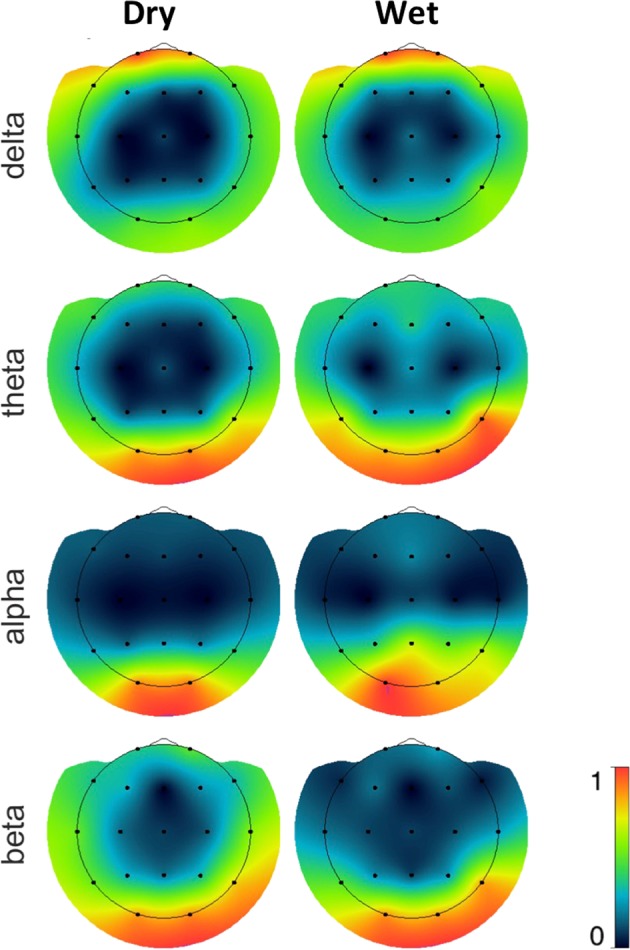
Spatial distribution of Delta2, Theta, Alpha and Beta power recorded by dry (left) and wet (right) electrodes, all referenced to the common average reference. Topographic plots were normalized within system.

In order to determine whether the dry EEG system would show similar spatial distribution of frequency band power as the wet EEG system, we compared the spatial distribution of the band power by means of a topographic map. For this purpose we averaged for both systems and each frequency band the absolute band power values over all subjects and all impedance ranges but separately for each channel. From these average values topographic maps were created by kriging which is a method to interpolate an irregularly-gridded 2D-set of points^44^. Finally the maps were individually scaled so that its minimum and maximum were mapped to the interval [0,1]. Due to this normalization the differences in absolute band power values between dry and wet recordings (see Table 3) are not visible. As shown in Fig. 7, the topographic maps for the dry EEG system resemble the corresponding maps for the wet EEG system.

#### Susceptibility to 50 Hz line noise

**Figure 8:**
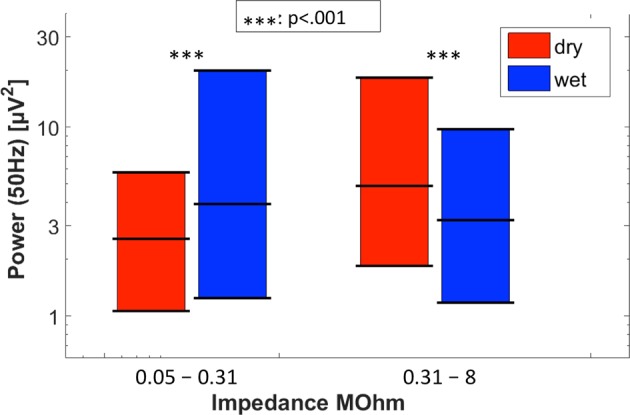
Spectral power around 50 Hz for the dry (red) and wet (blue) EEG systems. Left two bars: 25-/50-/75-percentiles observed during dry and wet electrode recordings at those electrode locations of all subjects where the dry electrode impedances were below the median (0.31 MOhm). Asterisk denotes significant difference between wet and dry EEG systems. Right two bars: Same as above, for impedances above the median. Asterisk denotes significant difference between wet and dry EEG systems.

As a general rule, power around line noise frequency increases with increasing electrode impedance^45^. Figure 8 shows the 25^th^, 50^th^ and 75^th^ percentiles of spectral power around 50 Hz (calculated over the frequency band 49–51 Hz) as observed for the wet and dry EEG systems setting for low and high impedances, i.e. impedance below or above the median impedance (0.310 MOhm). For lower impedances, the dry EEG system showed less line noise than the wet EEG system (mean/median/SD = 10.0/2.6/30.5 µV^2^ vs. 33.1/3.9/121.4 µV^2^; *Z* = −4.98, *p* = 0.0001), whereas at higher impedances the dry electrodes showed more line noise (45.0/4.9/189.0 µV^2^ vs. 11.1/3.2/21.4 µV^2^; *Z* = 3.36, *p* = 0.0008).

#### P3 ERP

**Figure 9:**
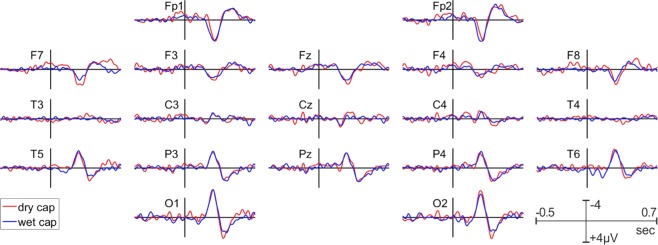
Grand average P3 waveforms for the dry (red) and wet (blue) EEG systems.

**Figure 10:**
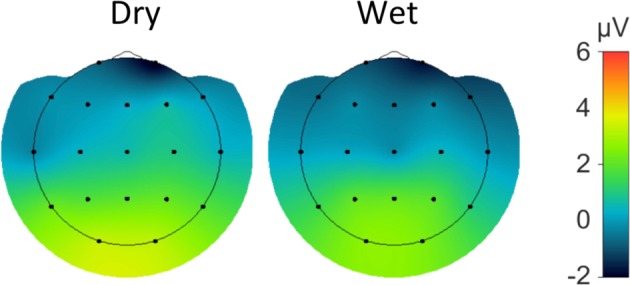
Spatial distribution of the grand average P3 for the dry (right) and wet (left) EEG systems.

Figure 9 shows the grand average difference waveform (target – standard) of the P3 ERP for all channels in both EEG systems. The peak latency of the P3 was comparable between the wet EEG system (396 ms) and dry EEG system (388 ms). The P3 amplitudes as observed at the O1/O2 electrode locations and the pre stimulus-baseline are shown in Tables 4 and 5 respectively. The P3 amplitude did not significantly differ between the wet and dry EEG systems. In contrast, the standard deviation of the pre-stimulus baseline was significantly higher for the dry than for the wet EEG recording. The topographical distribution of the P3 amplitude is comparable between the two systems, as shown in Fig. 10.

**Table 4:** Amplitudes of P3- and VEP-P1 components. SD = standard deviation.

ERP Amplitudes						
	Electrode location O1			Electrode location O2		
	Dry (Mean/median/SD)	Wet (Mean/median/SD)	Statistical comparison (Z/p)	Dry (Mean/median/SD)	Wet (Mean/median/SD)	Statistical comparison (Z/p)
P3 ERP -Peak	3.3/2.8/3.9 μV	2.7/2.8/2.7 μV	Z = 1.04, *p* = 0.29	3.2/2.5/3.4 μV	2.4/2.7/2.7 μV	1.31/0.19
P100 VEP -peak	4.9/4.3/3.6 μV	4.6/3.9/2.9 μV	−0.17/0.86	5.5/4.6/4.0 μV	5.4/4.4/4.1 μV	−0.15/0.88

**Table 5:** Pre-stimulus baseline standard deviation of the P3- and VEP-potentials. SD means standard deviation (i.e. the variability of the standard deviations computed separately for each electrode location).

	ERP Baseline-standard deviation, all electrodes		
	Dry (Mean/median/SD)	Wet (Mean/median/SD)	Statistical comparison (Z/p)
P3 ERP	2.2/2.0/0.73 μV	1.7/1.6/0.47 μV	3.31/0.0009
P100 VEP	0.43/0.41/0.14 μV	0.37/0.33/0.11 μV	2.38/0.02

In contrast to the often frontally prominent N2 component we here observed a more posterior distribution. However, several groups reported that the N2-topography substantially varies with task and stimulus features^46,47^. The ERPs observed over the FP1 and FP2 sites in response targets likely reflect the negative frontal slow wave often reported in P3 studies. The P3 is often absent or reduced over F3 and F4 sites as in our data and a frontal negativity emerges^48,49^. This is more evident in our fronto-polar data.

#### P100 VEP

**Figure 11:**
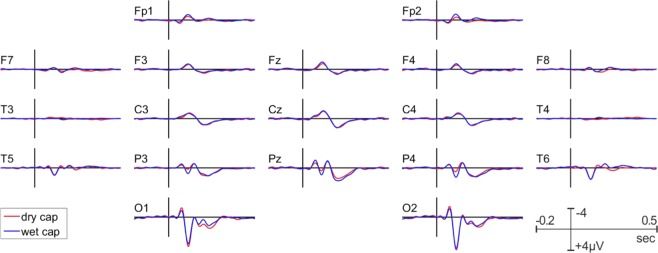
Grand average VEP-P100 waveforms for the dry (red) and wet (blue) EEG systems.

**Figure 12:**
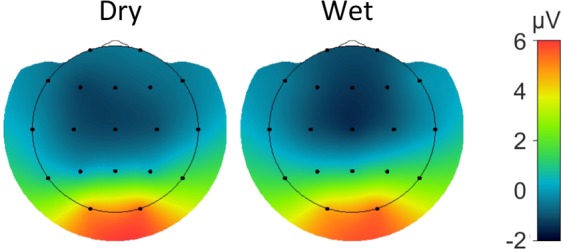
Spatial distribution of the grand average VEP-P100 for the dry (red) and wet (blue) EEG systems.

The grand average difference waveforms (target – standard) of the P100 are shown for all channels in both recording systems in Fig. 11. The peak latency of the P100 VEP was comparable between the wet (113 ms) and dry (114 ms) EEG systems. The P100 amplitudes as observed at the O1/O2 electrode locations and the pre stimulus-baseline are shown in Tables 4 and 5 respectively. The P100 amplitude did not significantly differ between the wet and dry EEG systems. In contrast, the standard deviation of the pre-stimulus baseline was significantly higher for the dry than for the wet EEG system. The topographical distribution of the P100 amplitude is comparable between the two systems, as shown in Fig. 12.

## Discussion

Multiple approaches have been proposed for dry electrodes usage in clinical and research settings see^1,2^. Most of the dry EEG systems used in these studies did not meet the clinical home monitoring requirements; for instance, inclusion of all electrodes in the10–20 montage or beyond, elimination of electrode caps with tight chin straps, wireless transmission plus optional local storage of long term recordings. Our results suggest that the dry and wireless F1 EEG system used in the current experiment is comparable in all dimensions to a conventional wet and wired EEG system.

Based on subjective reports, most subjects preferred the dry electrode headset (dry: 74%, wet: 10%). This is confirmed by the comfort levels reported by a second cohort of subjects wearing both headsets over one hour on different days. In particular, after 60 min the rating was primarily in favor of the F1 dry EEG headset thus indicating that this system may be considered as more acceptable by patients if recording periods exceed the clinical standard of 20 min, as it may occur in particular in home recording settings. In post-experiment interactions, subjects often reported that they preferred the absence of a chin strap in the dry EEG system, and they felt less ‘tied’ or restricted to the EEG amplifier as compared to the wet system. Absence of electrode gel was also reported as a positive feature of the dry EEG system. All of these factors are important considerations for EEG recordings in the context of home monitoring usage, repeated recordings during neurofeedback as well as during everyday activities, for instance practicing sports or monitoring vigilance. The dry electrode system afforded an advantage of only 2.5 min in set-up time, which means that that both systems can be set up in a timely manner.

Several groups have reported that signals recorded from dry electrodes are noisier^27,50–53^. This is consistent with our findings of a larger number of artifactual segments with the dry EEG system for both visual tasks. In addition, the greater power in-low frequencies (Delta and partly Theta, see Table 3) in the dry EEG system indicates underlying artifactual signal fluctuations. Notably, two experienced neurologists reported no differences in spontaneous EEG activity between dry vs. wet systems based on visual inspection. This conclusion is supported by all objective metrics included in our study that showed comparable performance between the two systems, including spectral power in clinically relevant frequency bands (i.e. Theta at low impedances, Alpha, and Beta), latency and amplitude of event related potentials (i.e. P100 and P3) as well as their topographical maps. Line noise in EEG recording systems is non-uniformly dependent on the magnitude of the impedances across all electrodes. Surprisingly, although impedances for the dry system exceeded the impedances observed for the wet system, the line noise level for the dry electrodes was lower than that for the wet electrodes. The only exception is when the dry electrodes’ impedance exceeded the median value (0.3 MOhm). Path length from the electrode to the amplifier or effectiveness of electrodes shielding may contribute to these differences.

Previous studies have examined the clinical application of different dry electrode head sets based on a 10–20 system. Slater *et al*.^54^ verified (i) that dry electrodes speeded data acquisition of interpretable EEG and (ii) demonstrated qualitatively that wet and dry electrodes were comparable by visual evaluation. Both findings are in line with our results from two experienced neurologists blinded to the EEG systems during evaluation. However, this study did not evaluate quantitative measures as well as artifact sensitivity. Halford *et al*.^50^ assessed practical aspects and the signal quality of spectral measures. They reported increased power in the dry recordings as compared to the wet recordings, in particular in lower (<4 Hz) frequencies and around 10 Hz. This increased power in lower frequencies (<4 Hz) was also observed in our data.

Our spectral results provide evidence that low frequency power (4–30 Hz) during resting state is comparable between the dry and wet recording systems. This confirms the clinical results of the visual evaluation by experienced neurologists, demonstrating that the minor difference in the Delta range (1.5–4 Hz) did not impair the clinical evaluation of these EEG signals. The comparable spatial distribution of all band power values provides further evidence that the eyes closed rsEEG acquired from the two systems are comparable. Accordingly, these results indicate our dry EEG system meets clinical standards.

P100 VEPs and P3 ERPs are two stimulus-evoked measures widely used in clinical settings and neurophysiology^34,55^. Given that dry, wireless electrodes are well suited for brain computer interface applications such as the P3 speller and motor control, it is important to determine whether the dry EEG system reliably records ERPs in the context of a clinical setting. Similar to the rsEEG results, both ERPs were almost identical in terms of their amplitude and latency recorded from the two systems. However, the pre-stimulus baseline variance is slightly larger with the dry electrodes system, possibly due to the higher impedance levels associated with the dry EEG system. Nevertheless, as shown in Figs. 9 and 11, the dominant ERP components are well delineated and there are no significant differences in the ERP latencies and amplitudes between the two recording systems. The spatial distributions of the P100 and the P3 (Figs. 9 and 11) are also well aligned. In summary, these results demonstrate that the dry and wireless F1 headset is suitable for acquisition of reliable event-related measures in a clinical context. From a clinical application point of view, this means that the dry electrode EEG system is capable of recording event related potentials that fully meet clinical needs.

## Conclusion

We directly compared dry and wet EEG systems based on the 10–20 electrode montage under clinical monitoring conditions that included both healthy volunteers and neurological patients with MCI. Although the number of artifacts was slightly higher for the dry EEG system and impedance values exceeded the typical 5 KOhm achieved with wet electrodes, the resting state EEG power and event-related potentials were comparable between the two systems. Importantly, both patients and healthy volunteers preferred the dry electrodes and reported that the dry headset was more suitable for self-application and potential home usage. Finally, the dry electrode headset is more robust to 50 Hz line noise indicating that it is less sensitive to electromagnetic interference from ambient noise one might encounter in the clinic or at home. Taken together, these results suggest the dry EEG system used in the current study has promise and should be further studied for its potential in home settings.
